# Role of Metastasis-Related microRNAs in Prostate Cancer Progression and Treatment

**DOI:** 10.3390/cancers13174492

**Published:** 2021-09-06

**Authors:** Su Jung Oh-Hohenhorst, Tobias Lange

**Affiliations:** 1 Martini-Klinik, Prostate Cancer Centre, University Medical Center Hamburg-Eppendorf, Martinistrasse 52, 20246 Hamburg, Germany; s.oh-hohenhorst@uke.de; 2 Institute of Anatomy and Experimental Morphology, University Cancer Center Hamburg, University Medical Center Hamburg-Eppendorf, Martinistrasse 52, 20246 Hamburg, Germany; 3 Centre de Recherche du Centre Hospitalier de l’Université de Montréal (CRCHUM) et Institut du Cancer de Montréal (ICM), Montreal, QC H2X 0A9, Canada

**Keywords:** microRNA, miRNA, non-coding RNA, prostate cancer, metastasis, epithelial-to-mesenchymal transition, EMT, biomarker, miRNA-based therapy, nanomedicine

## Abstract

**Simple Summary:**

In this review article we summarize the current literature on the pro- and anti-metastatic roles of distinct microRNAs in prostate cancer with a particular focus on their impact on invasion, migration and epithelial-to-mesenchymal transition. Moreover, we give a brief overview on how this knowledge developed so far into novel therapeutic approaches to target metastatic prostate cancer.

**Abstract:**

Prostate cancer (PCa) is one of the most prevalent cancer types in males and the consequences of its distant metastatic deposits are the leading cause of PCa mortality. Therefore, identifying the causes and molecular mechanisms of hematogenous metastasis formation is of considerable clinical importance for the future development of improved therapeutic approaches. MicroRNAs (miRNAs) are small non-coding RNAs that regulate gene expression at the post-transcriptional level by targeting messenger RNAs. Numerous studies have identified miRNAs as promotors or inhibitors of metastasis and revealed, in part, their targeting pathways in PCa. Because miRNAs are remarkably stable and can be detected in both tissue and body fluid, its potential as specific biomarkers for metastasis and therapeutic response is also currently under preclinical evaluation. In the present review, we focus on miRNAs that are supposed to initiate or suppress metastasis by targeting several key mRNAs in PCa. Metastasis-suppressing miRNAs include miR-33a-5p, miR-34, miR-132 and miR-212, miR-145, the miR-200 family (incl. miR-141-3p), miR-204-5p, miR-532-3p, miR-335, miR-543, miR-505-3p, miR 19a 3p, miR-802, miR-940, and miR-3622a. Metastasis-promoting RNAs, such as miR-9, miR-181a, miR-210-3, miR-454, miR-671-5p, have been shown to increase the metastatic potential of PCa cells. Other metastasis-related miRNAs with conflicting reports in the literature are also discussed (miR-21 and miR-186). Finally, we summarize the recent developments of miRNA-based therapeutic approaches, as well as current limitations in PCa. Taken together, the metastasis-controlling miRNAs provide the potential to be integrated in the strategy of diagnosis, prognosis, and treatment of metastatic PCa. Nevertheless, there is still a lack of consistency between certain miRNA signatures and reproducibility, which impedes clinical implementation.

## 1. Introduction

### 1.1. Prostate Cancer: State-of-the-Art

Prostate cancer (PCa) is the most frequently diagnosed cancer and the second leading cause of cancer-related death in males [1]. During the localized, early stage, PCa has outstanding survival rates thanks to surgery and radiation; advanced PCa with distant metastases, however, has a poor prognosis and dramatically reduced survival rates [2]. Thus, early detection of the individual metastatic potential and hence the identification of patients with potentially lethal forms of the disease is essential for clinical decision-making to improve patient outcome.

Despite the tremendous progress in diagnostic and therapeutic strategies during the last several years, metastatic progression is still responsible for most PCa patient deaths. Moreover, the incidence of metastatic PCa in the United States has risen over the past decade compared to reduced overall incidence [3].

Clinically, current classification is based on the Gleason score, prostate-specific antigen (PSA) level, and other clinical parameters that are still useful but not sufficient to predict metastatic potential, especially at the time of metastasis initiation. Tumors with similar histological patterns can lead to different clinical outcomes. The Gleason score may not exactly predict the aggressiveness and metastatic potential of the disease, similar to PSA levels, even though these parameters are powerful prognostic indicators [4,5,6]. Due to the lack of specific biomarkers for metastatic progression and due to the molecular heterogeneity of PCa [7], there is an urgent need for alternative biomarkers to refine PCa diagnosis, prognosis, and treatment response. Thus, there is a growing interest in the potential utility of microRNAs (miRNAs) in PCa tissue or serum as biomarkers that may open the way to personalized medicine [8,9].

### 1.2. miRNA

In recent decades, the improvements in sequencing technologies and bioinformatics resulted in the detection of a large number of non-coding RNAs (ncRNAs), including miRNAs, long ncRNAs, and circular RNAs [10,11]. MiRNAs are endogenous small non-coding RNAs (~22 nucleotides long) that are relatively stable in cells and body fluids, including plasma or serum [12,13,14,15]. Most miRNAs are transcribed from DNA into primary miRNAs (pri-miRNA), which are processed into precursor miRNAs (pre-miRNA) and finally into mature miRNAs. In most cases, miRNAs interact with the 3′ untranslated region (3′ UTR) of target messenger RNAs (mRNAs) to induce mRNA degradation and translational repression at the post-transcriptional level [16]. It is known that miRNAs control several important cellular processes, such as cell proliferation, cell cycle arrest, aging, and apoptosis [16,17,18].

Numerous studies have recognized cancer-related miRNA signatures with a highly aberrant expression that may initiate tumor growth and metastasis formation [17,18]. More specifically, the miRNA transcript profile in PCa was initially introduced by Porkka et al., who described 51 aberrantly expressed miRNAs in PCa using an oligonucleotide array hybridization procedure [19]. Among these, 37 miRNAs were down-regulated and 14 miRNAs up-regulated in PCa as compared to benign prostate tumors and hierarchical clustering by miRNA expression levels separated tumors according to their androgen dependence, suggesting miRNAs as novel diagnostic and prognostic tools in PCa [19].

As biomarkers, the specific molecular features of miRNAs have several advantages. First, miRNAs are remarkably stable and specific to be detected in formalin-fixed tissues [15], blood, and other body fluids because they are secreted into extracellular fluids as signaling molecules to mediate cell–cell communications [12,13,14,20]. In addition, expression levels and regulation of miRNA differ between different types of cancer [21] as well as individual tumors with different stages and molecular subtypes [22,23]. Therefore, specific miRNA profiles could be used to distinguish malignant from normal tissue and accurately recognize aggressive subtypes.

In recent years, abundant scientific efforts have been made to discover potential miRNA biomarkers and to understand their function in the initiation and metastatic progression of PCa [24,25]. Although current experimental methods are powerful enough to detect the abnormal change of miRNA expression of metastatic tumors versus primary PCa [24], it is still difficult to identify the key functional drivers due to the complexity of the metastatic cascade, the heterogeneity of the primary tumors (particularly in PCa), as well as the concurrent genetic and environmental factors contributing to disease progression [26,27].

With the help of bioinformatics analyses, molecular correlates between differentially expressed miRNAs and genes could be more easily identified. Zhu et al. have used public databases and miRNA sequencing data in order to identify and explore the relationships between differentially expressed miRNAs and genes [25]. In addition, they performed a pathway and process enrichment analysis and built a protein-protein interaction network and miRNA-mRNA regulatory network, enabling them to systematically define molecular signatures of bone metastasis in PCa. For instance, Zhu et al. identified miR-636 to be up-regulated in bone metastatic PCa tissue and found that this miRNA could promote migration and invasion by targeting MBNL2, TNS1 and STAB1. These target genes *(*MBNL2**, *TNS1* and *STAB1*) were also identified to have prognostic significance regarding biochemical recurrence (BCR)-free and disease-free survival of patients with PCa based on the data from The Cancer Genome Atlas (TCGA) [25]. Such computational approaches with high-throughput technologies are increasingly important for the identification of cancer-associated miRNAs and the prediction of the relationship between miRNAs and their target messenger [28,29] and will expedite the acquisition of new knowledge in this research field.

## 2. The Role of miRNAs in Migration, Invasion, and EMT in Prostate Cancer

More than 50% of miRNA genes are located in cancer-associated genomic regions [18]. They control the expression level of pro-metastatic genes by targeting mRNAs at the post-transcriptional level and therefore act as central nodal points for metastatic progression [30]. Because of the highly aberrant expression levels and, in some cases, aberrant sequences of miRNAs found in PCa [17,31], various studies have suggested the crucial regulatory role of miRNAs in PCa development and metastasis [24,30,32]. Moreover, a single miRNA can simultaneously target multiple mRNA genes; thus, even abnormalities of only a few miRNAs might significantly impact disease progression [9,10,33].

The formation of distant metastases, which are responsible for the majority of PCa-related deaths, is a highly complex process. One critical step in this process is the epithelial-mesenchymal transition (EMT) within the primary tumor that leads to the downregulation of epithelial cell characteristics and simultaneous upregulation of mesenchymal features [34]. EMT is regulated by transcription factors (EMT-TFs), which repress, for instance, the expression of E-cadherin (epithelial) and induce the upregulation of, e.g., N-cadherin (CDH2), vimentin, and fibronectin (mesenchymal). It is meanwhile widely accepted that the resulting downregulation of cell adhesion molecules leads to the spontaneous detachment of the future potentially metastatic cells from the primary tumor, their invasion across the basement membrane and migration through adjacent connective tissue at the early stages of metastatic spread [35,36].

The most widely described EMT-TFs are Snail-related zinc-finger transcription factors—Snail and Slug, ZEB1/2, and the Twist family (Twist1/2) [37,38]. The Snail and ZEB family of transcription factors are highly conserved zinc finger transcription repressors that bind to E-box-like promoter elements in DNA, thereby influencing target gene expression. The Twist family consists of a basic helix–loop–helix (bHLH) domain and a c-terminal Twist box for its transcriptional activity [39]. These EMT-TFs regulate the expression of various EMT- and tumor-related genes and are also associated with cancer progression and metastasis formation [40].

## 3. Signaling Pathways Involved in EMT

At least four fundamental regulatory networks orchestrate the EMT program: (i) transcriptional control, (ii) regulation from small non-coding RNAs, (iii) differential splicing, and (iv) translational and posttranslational control [41]. The best characterized regulatory network of the EMT process is transcriptional control, which involves the abovementioned EMT-TFs. The expression of these EMT-TFs can be activated through several signaling pathways. Transforming growth factor-beta (TGFβ), fibroblast growth factor receptors (FGFRs), and platelet-derived growth factor (PDGF) belong to the major players in this process, as well as other signaling pathways, such as Wnt, Notch, and nuclear factor (NF)-κB signaling, which induce EMT [36]. TGFβ is a prominent inducer of EMT and acts by targeting the EMT-TFs through Smad-dependent and -independent transcriptional pathways [42,43]. TGFβ also networks other EMT-inducing pathways [36] and their downstream effectors, such as PI3K and MAPK. NF-κB signaling regulates Snail, Slug, ZEB1/2, and Twist to repress epithelial phenotypes, either directly or indirectly, and induce mesenchymal markers like vimentin, MMP-2, and MMP-9.

Numerous studies have identified miRNAs as promotors or inhibitors of metastasis and revealed their targeting pathways, which primarily directly regulate EMT in PCa. Table 1 shows the EMT- and metastasis-regulating miRNAs in PCa and the involved signaling pathways.

## 4. Metastasis-Suppressing miRNAs in Prostate Cancer

### 4.1. miR-33a-5p

Dai et al. recently reported a negative correlation of ZEB1 expression and TGF-β signaling activity with miR-33a-5p expression in PCa [50]: downregulated miR-33a-5p expression in PCa tissues with bone metastases and bone-derived cells was positively correlated with advanced clinicopathological characteristics, shorter overall survival, and bone metastasis-free survival of the patients. Knockdown of miR-33a-5p promoted EMT, invasion, and migration of PCa cells, while up-regulating miR-33a-5p repressed the dissemination of PC-3 cells to the bone after intracardiac (left ventricle) injection into immunodeficient mice. Furthermore, miR-33a has been shown to target Engrailed-2 (EN-2), a homeobox-containing transcription factor, which was accompanied by suppressed proliferation, migration and invasion of human PCa cells [85].

### 4.2. miR-34

The well-known miRNA-34 family has been reported to be dysregulated in various cancers [86]. The miRNA-34 family comprises miR-34a, miR-34b, and miR-34c. miR-34a is located on chromosome 1p36, whereas, in the human genome, miR-34b and miR-34c are located on chromosome 11q23, clustered as homologous genes that share a common primary transcript [54]. The miR-34 family members are regarded as tumor-suppressive miRNAs due to their synergistic effect with the tumor suppressor p53, as well as their involvement in EMT via EMT-TFs. miR-34a inhibits metastasis by directly repressing CD44 [87], a cancer stem cell marker that has been considered to indicate a more mesenchymal phenotype of human PCa cells [88]. Moreover, miR-34a represses WNT/β-catenin, JAK/STAT3, and PI3K/AKT signaling, all of which have been related to PCa progression [54,86,87]. miR-34a also down-regulates the oncoprotein STMN1 through the CtBP1\miR-34a\STMN1\GDF15 axis and prevents PCa progression in vivo [89]. MiR-34b similarly carries out its migration- and invasion-inhibiting role by regulating the TGF-β pathway via phosphorylation of SMAD3 (TGF-β/SMAD3 pathway) in metastatic PCa [54]. In a recent study from our group, miR-34 is also included in a metastasis-related interaction network of PCa, in which miR-34 expression is down-regulated with increasing metastatic potential of spontaneous metastasis xenograft models. In the same network, E-cadherin is concurrently down-regulated and N-cadherin, Sparc and Slug are up-regulated with increasing metastatic competence [65]. Together with miR-34, the miR-15a/16 cluster was also down-regulated within the network, which is supported by a former study proposing miR-15a/16 as tumor suppressor genes in PCa that control cell proliferation, survival and invasion [90].

### 4.3. miR-132 and miR-212

Experimental overexpression of miR-132/212 was reported to inhibit TGFβ-induced EMT in human PCa cells, accompanied by less migratory and invasive behavior in vitro. This effect was proposed to be mediated via SOX4, an important EMT regulator in PCa [55]. In a meta-analysis, downregulation of miR-132 was shown to be associated with an increased relative risk of distant metastasis; in contrast to the aforementioned study, however, the same meta-analysis revealed that high (but not low) levels of miR-212 are associated with increased risk of distant metastasis in PCa [91]. Therefore, the exact role of miR-212 for metastatic progression in PCa remains to be clarified.

### 4.4. miR-145

miR-145 is activated by p53 and inhibits mesenchymal and cancer stem cell markers such as fibronectin, vimentin and CD44 [56]. miR-145 also blocks c-Myc by targeting two oncogenic mediators, human enhancer of filamentation 1 (hEF1) and Krüppel-like factor 4 (KLF4) [56,57]. miR-145-5p and miR-145-3p are targets of metadherin (MTDH) [92] which is highly expressed in several tumors. In PCa, both these miRNAs were found to suppress the growth and metastasis of PCa cells by negatively regulating the expression of MTDH. Based on the oncogenic function of MTDH and its inhibition by miR-145-5p or miR-145-3p, the miR-145-5p/MTDH and miR-145-3p/MTDH pathways provide potential therapeutic targets for miRNA-based therapy of PCa [58,59].

### 4.5. miR-200 Family

The miR-200 family consists of five members (miR-200a, -200b, -200c, -141, and -429) and two clusters located on chromosome 1 and 12 [93]. Members of the miR-200 family, which are commonly downregulated in several types of cancer, target different signaling pathways, including Notch, Wnt, and TGF-β. Hence, the loss of the miR-200 family in cancer contributes to the migratory and adhesive potential of tumor cells, to the EMT program, and angiogenesis [94]. For instance, the miR-200 family directly targets the snail family transcriptional repressor 2 (SNAI2/SLUG) to inhibit EMT [70]. Despite a well-documented role of the miR-200 family in ovarian, lung, renal, and breast cancer [93], only a few studies are available in the field of metastatic PCa. Accordingly, miR-200 family members were not included in a recently published metastasis-related mRNA/miRNA interaction network that was based on differentially metastatic spontaneous metastasis PCa xenografts [65].

Nevertheless, miR-200a and miR-200b have been previously identified as androgen-regulated miRNAs, and miR-200a has been shown to suppress the development of castration-resistant prostate cancer (CRPC) by impairing BRD4-mediated AR signaling [95]. Likewise, miR-200b has been identified as a downstream androgen receptor target, and its expression has been associated with decreased tumorigenicity, EMT induction, and metastatic competence of PCa cells [96]. The effect of miR-200c on invasion and migration has been studied mainly at the in vitro level [97]. In particular, miR-200c was shown to be downregulated in *TMPRSS2: ERG*-fusion-positive PCa [69]. Kim et al. investigated how ERG-mediated suppression of miR-200c activates *ZEB1* expression, which is responsible for inducing the EMT program. The authors reported miR-200c as the first miRNA target of ERG and suggested that miR-200c could be used therapeutically in PCa patients harboring the *TMPRSS2*: *ERG* gene fusion. All members of the miR-200 family can be detected in the serum of CRPC patients, with high expression levels indicating an unfavorable prognosis [68,71].

miR-141-3p also belongs to the miR-200 family. The clinically reduced expression of miR-141-3p in bone metastatic PCa tissues compared with non-bone metastatic PCa suggested a metastasis-suppressive role of miR-141-3p. In vitro, upregulation of miR-141-3p suppresses EMT, as well as invasion and migration of PCa cells, and dramatically reduces dissemination of PCa cells to the bone after intracardiac (left ventricle) injection, while silencing this miR yields an opposite effect. miR-141-3p is negatively correlated with NF-κB signaling and inhibits its activation by directly targeting tumor necrosis factor receptor-associated factor 5 (TRAF5) and 6 (TRAF6) [61]. In a meta-analysis, down-regulation of miR-141-3p was confirmed to be associated with higher risk of distant metastasis in PCa [91].

### 4.6. miR-204-5p

miR-204-5p suppresses invasion, migration, and dissemination of PCa cells to the bone after intracardiac injection through deactivation of nuclear factor κB (NF-κB) signaling by simultaneously targeting TRAF1, TAB3, and MAP3K3 [72]. In clinical PCa samples, miR-204-5p expression negatively correlates with TRAF1, TAB3, and MAP3K3 expression and NF-κB signaling activity. In an independent cell line-based study, miR-204 was found to be among different miRs that have a major effect on gene dysregulation in PCa [22].

### 4.7. miR-532-3p

Like most of the so far mentioned suppressor miRNAs, a metastasis-suppressive role was also suggested for miR-532-3p based on reduced expression levels in bone metastatic PCa tissues. Similar to miR-141-3, miR-532-3p inhibited activation of nuclear factor κB (NF-κB) signaling via simultaneously targeting TRAF1, TRAF2, and TRAF4. Upregulation of miR-532-3p inhibited invasion, migration, and dissemination of PCa cell to the bone after intracardiac injection [75]. These preclinical investigations suggest a role of miR-532-3p for suppressing bone metastasis in PCa.

### 4.8. mirR-335 and -543

miR-335 and -543 have also been associated with bone metastasis in PCa by affecting their common target, i.e., endothelial nitric oxide synthase (eNOS) [76]. Fu et al. first identified four miRNAs (miR-335, -543, -196, and -19a) that are significantly downregulated in PCa bone metastases compared with PCa primary tumors. Downregulation of miR-335 and -543 was confirmed in a total of 20 paired samples from primary PCa and bone metastases. Simultaneous upregulation of mRNA expression and protein levels of eNOS was also demonstrated in these samples. Furthermore, exogenous overexpression of miR-335 and -543 markedly downregulated the expression level of eNOS and significantly impaired the migratory and invasive ability of PCa cells in vitro. An independent PCa study demonstrated that low expression of miR-335 was significantly associated with a higher Gleason score, advanced tumor stage and the presence of metastases [98].

### 4.9. miR-505-3p and miR-19a-3p

miR-505-3p and miR-19a-3p have the same targets and inhibit the invasion and migration abilities of PCa cells via targeting downstream effectors of TGF-β signaling, SMAD2 and SMAD4, and thereby deactivate TGF-β signaling. These reports were based on preclinical investigations [77,78].

### 4.10. miR-802

Experimental overexpression of miR-802 was reported to suppress EMT, migration and invasion of human PCa cells in vitro and to delay xenograft tumor formation in vivo. These effects were proposed to be mediated through targeting of flotillin-2 [80], a caveolae-associated, integral membrane protein that tethers growth factor receptors linked to signal transduction. In a meta-analysis, downregulation of miR-802 was confirmed to be linked to increased relative risk of distant metastasis in PCa [91].

### 4.11. miR-940

Rajendiran et al. reported that miR-940 suppresses PCa migration and invasion by regulating migration and invasion enhancer 1 (MIEN1) [82]. Analysis of human PCa and associated normal glands confirmed a higher expression of miR-940 in normal tissues than in the tumors. MIEN1, located next to HER2/*neu* in the 17q12 locus, was a direct target of miR-940. MiR-940 inhibited the downstream effectors of MIEN1 and increased E-cadherin expression, suggesting its role in mesenchymal-to-epithelial transition (MET) as studied in PCa cell lines. More recently, however, Rajendiran and coworkers found higher miR-940 levels in the serum of PCa patients and proposed it as a potential serum marker for PCa diagnosis [81]. Considering their previous study, the authors hypothesized that miR-940 might be exported from cancer cells to prevent the downregulation of MIEN1 and other predicted target proteins that promote cancer progression. Moreover, an independent group demonstrated that miR-940 is highly expressed in exosomes released from PCa cell lines (C4, C4-2 and C4-2B) that induce an osteoblastic phenotype, in contrast to cell lines that induce an osteolytic phenotype, such as breast cancer (MDA-MB-231) and myeloma cell lines (KMS11, U266). Accordingly, miR-940 promoted osteogenic differentiation of human mesenchymal stem cells [83]. Therefore, secreted miR-940 may be be functionally involved in the formation of bone metastases in PCa, which typically show an osteoblastic phenotype.

### 4.12. miR-3622a

Bucay et al. have demonstrated that endogenous miR-3622a expression is crucial to maintain the epithelial features of healthy prostate cells [84]. MiR-3622a inhibited EMT, progression, and dissemination of PCa in vitro and in vivo by directly targeting the EMT-TFs ZEB1 and SNAI2. The authors assumed that the frequent loss of miR-3622a at the chr8p21 region leads to the induction of EMT states and is responsible for PCa progression and metastasis.

## 5. Metastasis-Promoting miRNAs

### 5.1. miR-9

Based on laser-capture microdissected radical prostatectomy specimens, miR-9 was found to be overexpressed in PCa tissue compared to adjacent benign glandular epithelium [44]. In this study, miR-9 was demonstrated to target E-cadherin so that inhibition of miR-9 led to reduced migratory and invasive potential of PCa cells. These findings are strongly supported by our own observation that miR-9 monotonically increases with rising metastatic potential of spontaneous metastasis PCa xenografts, accompanied by monotonically decreasing E-cadherin (and desmoplakin) expression [65].

### 5.2. miR-181a

miR-181a was demonstrated to promote EMT in PCa by targeting TGIF2 [62]. Zhiping et al. currently observed an up-regulation of miR-181 in metastatic PCa samples. This expression profile was consistent with the preclinical observation that overexpression of miR-181a promoted PCa cell migration, invasion and EMT. TGIF2, a repressor of the Smad pathways was identified as direct target of miR-181a in PCa cells (Smad 2/3 activation induces EMT). These findings described a miR-181a-TGIF2-Smad-EMT axis in metastatic PCa cells and provide a rationale for a miR-181a targeting strategy as novel therapeutic approach to treat advanced PCa. Moreover, miR-181a-5p could be isolated from the serum of PCa patients suffering from bone metastases and was suggested as promising diagnostic biomarker for metastatic PCa [63].

### 5.3. miR-210-3

miR-210-3 was shown to promote EMT by constitutive activation of NF-κB signaling [73]. The expression of miR-210-3 correlated with the bone metastasis status in PCa patients as well as with SOCS1 and TNIP1 signaling activity, which was verified in metastatic PCa tissues. While upregulated miR-210-3p enhanced EMT, invasion and migration of PCa cells, silencing miR-210-3p repressed dissemination to the bone marrow in vivo. In an independent meta-analysis, miR-210-3 was found to be associated with higher relative risk for distant metastasis [91].

### 5.4. miR-454

miR-454 is highly expressed in PCa and targets the well-known tumor suppressor N-myc downstream-regulated gene 2 (NDRG2) which consequently induces proliferation and invasion of PCa cells [74]. miR-454 was also shown to activate WNT/ß-catenin signaling which is able to interact with Ras so that miR-454 might contribute to bone metastasis in PCa via these pathways [99]. Its potential as therapeutic target is consistently suggested [100], but not well-validated in PCa yet.

### 5.5. miR-671-5p

Zhu and colleagues recently reported a metastasis-promoting action of miR-671-5p [79]. They initially identified 13 miRNAs related to PCa metastasis by bioinformatics analysis. Amongst them, miR-671-5p was increased in metastatic PCa tissues and its expression was associated with poor prognosis of PCa patients. In preclinical investigations, miR-671-5p promoted migration and invasion of PCa cells by suppressing the NFIA/CRYAB axis. In clinical analyses, high expression of NFIA and CRYAB in PCa tissue negatively correlated with advanced clinicopathological characteristics and a positive metastasis status of PCa patients, supporting a metastasis-promoting role of miR-671-5p.

## 6. Metastasis-Related miRNAs with Conflicting Evidence

### 6.1. miR-21

mir-21 is one of the first miRNA found upregulated in a variety of cancers, such as glioma, breast cancer, and colorectal cancer and thereby considered as oncogenic miRNA in PCa. PTEN (phosphatase and tensin homolog), RECK (reversion-inducing cysteine-rich protein with Kazal motifs) and PDCD4 (programmed cell death protein 4), which are linked to decreased metastatic potential and found dysregulated in PCa, belong to the target genes of miR-21 [101,102,103,104]. STAT3 (signal transducer activator of transcription 3), a transcription factor activated by IL6 in PCa, was also shown to interact with miR-21 [105]. KLF5 was also identified as target and inhibited by miR-21 at both mRNA and protein level [48]. Increased PCa cell migration and invasion in vitro as well as xenograft tumor growth by overexpression of miR-21 were confirmed [48,101]

Consequently, miR-21 has been proposed as a potential diagnostic biomarker, as well as a therapeutic target for PCa [49,106]. However, its potential as a biomarker should be viewed with caution due to some controversial observations [45,46,47] The lack of reproducibility could be attributed to the diversity of patient cohorts, sample processing, measurement techniques and data analysis [107,108].

### 6.2. miR-186

There are several controversial reports about the expression of miR-186 in PCa and its role in metastatic progression. miR-186-5p was found to be up-regulated in the serum of patients diagnosed with PCa as compared to healthy individuals and inhibition of this miRNA reduced invasion of PCa cells in vitro [64,109]. In the aforementioned xenograft model-based, metastasis-related miRNA/mRNA interaction network, miR-186 was also monotonically up-regulated with rising metastatic potential. One of the identified targets of miR-186 was the membrane cofactor protein CD46, whose loss was demonstrated to indicate shortened biochemical relapse-free survival in multivariate analyses [65]. By contrast, Zhao et al. identified down-regulated miR186 expression in human PCa specimens, most significantly in the metastatic patient specimens [66] and suggested miRNA 186 as a metastasis suppressor in PCa. The low miR186 expression was also correlated with poor patient survival which supported metastasis-suppressing properties. The enforced overexpression of miR186 inhibited cell motility, invasion, colony formation, and vasculogenic mimicry (VM) formation capacity, as well as the EMT process by targeting Twist1 in vitro and in vivo and suggested a therapeutic potential of miR186/Twist1. A similar preclinical investigation also reported down-regulated miR-186 in PCa cell lines in comparison to a benign cell line (RWPE-1) and identified YY1 and CDK6 as additional targets of miR-186 for their tumor-inhibitory action [67].

Although the majority of studies verified miR-186 as a tumor suppressor miRNA, the conflicting findings on its oncogenic role in PCa may impede the application of miR-186 as a diagnostic and therapeutic target [110]. A possible dual role of miR-186 in the metastatic process of PCa should be clarified by extended further research with improved study protocols.

## 7. Therapeutic Approaches Based on miRNA Regulation

The identification of the various roles of miRNAs in human disorders led to the development of miRNA-based treatment strategies providing several molecular advantages. In particular, agonists or antagonists of a single miRNA can achieve strong effects through the regulation of multiple pathways, because each miRNA regulates the expression of several target genes which are also able to enhance each other. Furthermore, miRNAs can be detected in differently processed patient tissues as well as in body fluids such as serum or urine. Such properties of miRNAs suggest a great therapeutic and biomarker potential for PCa diagnosis, prognosis and treatment.

The therapeutic approaches that are based on miRNA regulation include the use of miRNA mimic synthetic products to target the 3′-UTR of the targeted oncogenes, antisense oligonucleotides-anti-miRNAs (anti-miRs) or synthetic constructs to reduce the levels of intracellularly overexpressed miRNAs and compounding miRNA-based tools with conventional anticancer therapeutics.

Basically, miRNA mimic synthetic products for cancer therapy act like the tumor-suppressive miRNAs. miRNA mimics are constructed to replace the lost or downregulated tumor-suppressive miRNA in tumor tissue and target potentially overexpressed oncogenes. The other way around, anti-miRs inhibit the tumor promoting miRNAs via direct binding to the small RNA species within the RNA-induced silencing complex and reverses their downstream effects, resulting in decreased tumor burden [100]. However, an effective delivery of such products into target tissues is still a challenge.

Two main strategies, i.e., intra-tumoral (local) or systemic delivery, have been considered for the application of miRNA mimics or anti-miRs. The local delivery may selectively deliver therapeutic miRNAs into target tissues without impairing the non-tumor tissues, but is not suitable for metastatic cancer. Therefore, significant efforts have been made to develop systemic miRNA delivery strategies. Preclinical evaluations of various therapeutic approaches based on miRNA in PCa are summarized in Table 2.

The replacement of tumor-suppressive miRNA can be attempted by viral vectors expressing corresponding miRNA, nanoparticles and small compounds that regulate the endogenous expression of miRNA [100].

Takeshita and colleagues (2010) introduced systemic delivery of synthetic miR-16 using atelocollagen [111], a highly biocompatible material displaying an effective drug delivery technology for in vivo models [121]. Atelocollagen was shown to efficiently deliver synthetic miR-16 to tumor cells in bone tissues when injected into the tail vein of mice and inhibited the local growth of prostate tumors via downregulation of multiple cell-cycle genes such as CDK1 and CDK2.

Tang and colleagues (2018) introduced the agomiR-133a-3p, a synthetic tumor suppressor miRNA [114]. Transfection of 22Rv1, C4-2B and PC3 cells with agomiR-133a-3p resulted in targeting of multiple cytokine receptors such as EGFR, FGFR1, IGF1R and METa and led to inactivation of PI3K/AKT signaling. Based on the observation that the i.v. application of agomiR-133a-3p significantly reduced bone metastasis in a PC3 xenograft model, the authors suggested the systemic delivery of agomir-133a-3p as a potential anti-bone metastasis therapeutic strategy in PCa.

The use of miR-155-5p mimics was also investigated in an in vitro study [118]. The miR-155-5p mimics inhibited the expression of testican-1 (SPOCK1) in PC3 cells and decreased the cell’s ability to migrate and invade, while upregulating the expression of vimentin, N-cadherin, E-cadherin, β-catenin, matrix metalloproteinase (MMP) 3 and MMP9. Based on their analyses, the authors proposed SPOCK1 to be a direct target gene of miR-155-5p, a tumor suppressor in PCa. In addition, they showed that SPOCK1 promotes PCa invasion and migration [118].

Synthetic miRNA mimics may also increase chemosensitivity and may have therapeutic potential in CRPC by regulating genes involved in taxane response or resistance [122]. To identify a potential target for this purpose, Lin et al. performed a genome-wide screen of 1280 miRNAs in PC3 and DU145 cells in combination with docetaxel or cabazitaxel treatment, identifying miR-217 and miR-181b-5p as therapeutic targets [120]. Transfection with both miR-217 and miR-181b-5p mimics enhanced apoptosis in PC3 cells in the presence of these taxanes and downregulated more than 1000 different transcripts enriched for genes with cell proliferation and focal adhesion functions [120].

Different potential nanoparticles such as lipid-, cationic polymer- and peptide-based vectors have also been considered for miRNA delivery strategies [123,124]. Amongst others, the cationic polymer polyethyleneimine (PEI) demonstrated good biocompatibility and high transfection efficacy and was suggested as suitable vector for this purpose [125]. In a preclinical study with a PC3 xenograft mouse model, PEI has been used to replace the tumor suppressor miR-143 [116]. Although the significant tumor growth inhibition by PEI-miR-143 has confirmed the usefulness of PEI as a miRNA delivery system, its clinical applications are still limited due to the lack of specificity, and interaction with proteins in biological fluids, resulting in high cytotoxicity and tendency to aggregate following blood injection. To overcome these limitations, modifications of PEI and optimization of parameters of PEI-based formulations have been introduced [59,113,119].

A recent study reported such chemically modified PEI to replace the tumor suppressor miR-145 [59]. As previously mentioned, the metastasis-suppressive miR-145-5p/MTDH and miR-145-3p/MTDH pathways belong to the potential treatment targets for PCa. Poly-arginine (R11), a novel cell-permeable peptide represents a potential delivery vehicle facilitating an organ-specific uptake in PCa cells. The R11-labeled, chemically modified polyethyleneimine (R11-SSPEI) nanocarrier exhibited optimal transfection efficacy through electrostatic interaction of R11-SSPEI with miR-145. In a peritoneal mouse tumor model, systemic administration of the R11-SSPEI/miR-145 complex led to effective delivery of miR-145 into PCa cells and dramatically inhibited tumor growth, thereby prolonging the survival of the tumor-bearing mice [59].

Nagesh et al. introduced a novel magnetic nanoparticle (MNP)-based platform composed of an iron oxide core with PEI-poly (ethylene glycol) layer(s) (PEI-PEG) [119]. These optimized nanoparticles were able to escape the endo/lysosomal pathway and released intact miR-205 into cytoplasmic compartments. They demonstrated that the applicated miR-205 nanoplatform induced significant apoptosis of C4-2 and PC3 cells and enhanced the docetaxel-based chemotherapeutic effects in PCa cells.

Similarly, miR-124 mimics conjugated with PEI-functionalized polyhydroxybutyrate nanoparticles (PHB-PEI) have been reported to show antitumor activity in PCa cells [113]. miR-124 is a tumor suppressor and modulator of carnitine palmitoyltransferase 1A (CPT1A), a key enzyme of mitochondrial fatty acid oxidation which is important for cancer survival and activation of oncogenic pathways. PHB-PEI-miR-124 effectively protected miR-124 from RNAse degradation, resulting in increased delivery efficiency of intact miR-124 into PC3 cells. This nanoparticle-delivered miR-124 decreased CPT1A expression, impairing hallmarks of tumorigenicity, such as cell proliferation and motility in PC3 cells.

For the combined delivery of tumor-suppressive miR-146a and cetuximab, modified nano vectors made of PEI-PBA and PEI-DMA were engineered [117]. Both miR-146a and cetuximab are known to target EGFR. These nanoparticles contain an inner core polyplex (PEI-PBA-miR-146a) bound to the outer layer polyplex (PEI-DMA-cetuximab) via electrostatic interactions and escape the endo/lysosomal pathways protecting miR-146a from enzymatic degradation. While miR-146a-mediated EGFR silencing and reduced cell growth, invasion, and migration of DU 145 cells, the miR-146a- and cetuximab-loaded nanocomplexes strongly inhibited DU145 tumor growth in vivo [117].

Gauer et al. (2015) reported a different nanoparticle technology-mediated replacement of miR-34a, a tumor suppressive miRNA [112]. The chitosan nanoparticle-mediated delivery of miR-34a induced combined effects of autophagy and apoptosis by downregulation of MET, Axl and c-Myc, and non-canonical autophagy. The reduced prostate tumor growth and preservation of bone integrity in an intra-femoral PCa xenograft suggested that miR-34a may mediate tumor-suppressive effects by targeting both the tumor as well as the bone microenvironment.

Another promising targeting approach is the manipulation of overexpressed oncogenic miRNAs using antisense oligonucleotides-anti-miRNAs (anti-miRs) in order to reverse their downstream effects and decrease metastatic burden [100]. However, as in the case of synthetic miR mimic products, the sufficient delivery of anti-miRs presents a major challenge.

Most recently, Kim et al. demonstrated the anti-cancer potential of anti-miR21, which blocks the tumor suppressor PTEN. They presented the modified locked nucleic acid (LNA) and poly nucleic acid (PNA)-type anti-miR21 and its therapeutic efficacy in vitro and in a murine PCa model, which showed reduced bone metastasis formation upon treatment [106]. Although miR-21 is thought to possess oncogenic properties due to its ability to negatively modulate PTEN and its knockdown reversed the malignant phenotype in several tumor models, the therapeutic potential of miR-21 for PCa is still questionable due to conflicting literature. For instance, Folini et al. investigated the role of miR-21 and its potential as a therapeutic target in two PCa cell lines as well as in prostatectomy specimens characterized by different miR-21 expression levels and PTEN gene status [126]. They found that miR-21 was not differentially expressed in PCa and matched normal tissues from patients, and suggested that miR-21 may not be a central player in the development of PCa and that its targeting alone should not be a valuable therapeutic strategy.

Kunz et al. introduced further potential nanoparticle-complexed anti-miRs for inhibiting tumor growth and metastasis in PCa [115]. As therapeutic targets in PCa, oncogenic miR-141 and miR-375 were selected in this treatment study since both miRNAs have been found overexpressed in PCa patients [46]. PEI-complexed antimiR-141 and antimiR-375 reduced tumor growth of PCa cells in vitro and in vivo. The transfection of anti-miR-141 and anti-miR-375 induced a substantial reduction in cell proliferation and increased apoptosis in LNCaP, PC3 and DU145 cells. Likewise, intraperitoneal administration of specific PEI/anti-miR complexes in PCa xenografts led to an about 60% inhibition of primary tumor growth.

Despite numerous investigations in preclinical models of cancer, only a few miRNA candidates have reached clinical development so far. The first tumor-targeted miRNA drug, MRX34, based on miR-34a mimics and nano-scaled liposomal delivery systems was under clinical evaluation (NCT01829971) in hepatocellular carcinoma and metastasis from kidney, breast and lung cancer [86], but was halted due to side effects and its therapeutic efficiency has not yet been investigated in PCa.

Nevertheless, the therapeutic approach based on miRNAs for PCa is definitely an attractive and growing area of cancer research. While some miRNA-based therapeutics have already been under clinical evaluation in breast, lung, liver and kidney cancer, most PCa-related studies were performed at the preclinical level and some of them even reported inconclusive results. Therefore, a better understanding of the high complexity of miRNA-mediated gene regulatory networks is primarily required for the development of miRNA-based therapies in PCa. In addition, improved delivery systems and minimizations of therapeutic doses and off-target effects are crucial for a feasible therapeutic strategy of miRNA drugs.

There is currently a great hope in the field of RNA-based therapy and rapidly developing nanotechnology due to the first messenger RNA-based anti-COVID-19 vaccines with optimized nanoformulations for the efficient packaging and safe delivery of genetic material. This sensational event in nanomedicine could arouse more interest in miRNA-related cancer research and accelerate the development of the nanocarriers for miRNA therapeutics [127].

## 8. Conclusions

The significance of miRNAs for the metastatic progression of PCa and their potential as biomarkers is of growing clinical importance. The recently identified metastasis-controlling miRNAs provide the potential to be integrated in the strategy of diagnosis, prognosis, and treatment of metastatic PCa. Since it was discovered that miRNA could be detected in both body fluid and tissue, and nanoparticle-based delivery methods of synthetic therapeutic miRNAs has been introduced, their clinical use as biomarkers and therapeutic approaches is becoming one of the main focuses of current research.

Despite a growing number of published data and encouraging investigations in this field, most evidence has been obtained from preclinical studies. Moreover, there is still a lack of consistency between certain miRNA signatures and reproducibility, which additionally impedes its clinical implementation. These limitations may be due to the diversity of sample types (primary tumor and metastatic tissue, blood, urine and cell lines), patient cohorts and technological methods, as well as the lack of standardized protocols for sample handling and data analysis.

Therefore, further studies are required prior to implementing miRNA-based cancer diagnostic and therapeutic strategies into clinical practice. In this respect, extended research on experimental and analytical aspects are crucial to increase the translational impact of miRNA-related research. Furthermore, the development of effective strategies for delivering synthetic therapeutic miRNAs to desired target tissues may enhance the efficacy of miRNA-mediated treatments, allowing this type of therapy to be used in disease management in the future.

## Figures and Tables

**Table 1:** miRNAs involved in EMT and metastasis of prostate cancer.

miRNA	Expression in PCa	Function	Targets and Signaling Pathways	References
miR-9	Up-regulated in PCa tissue	promotes invasion and migration, increases spontaneous metastasis in vivo	E-cadherin	[44]
miR-21	Up-regulated in PCa tissue [45]	promoting invasion and migration in vitro, and xenograft tumor growth	PTEN, RECK. PDCD4, BCL-2, EGFR, KLF5	[45,46,47,48,49]
	Down-regulated in PCa tissue, serum, plasma, urine [46,47]			
miR-33a-5p	Down-regulated in bone metastatic PCa tissue and bone-derived cells (vs. non-metastatic PCa tissue)	inhibits EMT, migration and invasion and suppresses bone metastasis in vivo	Inactivation of TGF-β signalling via directly targeting transforming growth factor beta receptor 1 (TGFBRI)	[50]
miR-34 family	Down-regulated in PCa tissue	inhibits migration, invasion and increases metastatic potential in vivo	Repressing of CD44 and deactivation of WNT/β-catenin, JAK/STAT3, PI3K/AKT (miR34a)	[51,52,53,54]
			Targeting oncoprotein STMN1 through the CtBP1\miR-34a\STMN1\GDF15 axis (miR-34a)	
			Targeting TGF-β-pathway via phosphorylation of SMAD3 (miR-34b)	
miR-132 and miR-212	Down-regulated in PCa tissue	inhibits EMT, invasion and migration	Deactivation of TGF-β signaling via SOX4	[55]
miR-145	Down-regulated in PCa tissue and serum (CRPC)	inhibits invasion, migration and arrests cell cycle	Inhibition of c-Myc via targeting hEF1 and KLF4	[56,57,58,59,60]
	Up-regulated in urine (Xu et al., 2017)		Inhibition of MTDH	
miR-141-3p	Down-regulated in bone metastatic PCa tissue	suppresses EMT, invasion and migration and dissemination of tumor cells	Deactivation of NF-κB signaling by targeting TRAF5 and TRAF6	[61]
miR-181a	Up-regulated in metastatic PCa vs. localized PCa tissue and serum (bone metastatic PCa)	promotes migration, invasion and EMT	SMAD2/3 activation by targeting TGIF2	[62,63]
miR-186	Up-regulated in PCa tissue, PCa xenograft, and serum(186-5p) [64,65]	enhances colony formation, 3D culture growth, vasculogenic mimicry (VM) formation capacity, invasion, and EMT	Twist1, membrane cofactor protein CD46	[64,65]
	Down-regulated in primary and metastatic PCa tissue, PCa cell lines vs. control [66,67]		YY1 and CDK6	[66,67]
miR-200 family	Down-regulated in TMPRSS2: ERG-fusion pos. PCa (miR-200c) and in bone metastatic PCa tissue (vs. localized PCa) (143-3p) [68]	inhibits migration, angiogenesis, cell adhesion and EMT	*SNAI2/SLUG*, *ZEB* and deactivation of Notch, Wnt, and TGF-β signaling	[61,69,70,71]
			Deactivation of NF-κB signaling by targeting TRAF5 and 6 (143-3p)	
miR-204-5p	Down-regulated in PCa tissue	suppresses invasion, migration, and dissemination of tumor cells	Deactivation of NF-κB signaling by targeting TRAF1, TAB3, and MAP3K3	[72]
miR-210-3p	Up-regulated in bone metastatic PCa tissue (vs. non-metastatic PCa)	enhances EMT, invasion and migration and bone metastasis in vivo	activation of NF-κB signaling via targeting TNIP1 and SOCS1	[73]
miR-454	Up-regulated in in PCa tissue and cell lines	promotes PCa cell proliferation and invasion	Promoting N-myc downstream-regulated gene 2 (NDRG2) and inhibition of WNT/β-catenin signaling	[74]
miR-532-3p	Down-regulated in PCa tissue with bone metastasis	inhibits invasion, migration, and dissemination of tumor cells	Deactivation of NF-κB signaling via targeting TRAF1, TRAF2, and TRAF4	[75]
mirR-335 and -543	Down-regulated in primary PCa tissue and bone metastasis	inhibits migration and invasion	Targeting endothelial nitric oxide synthase (eNOS)	[76]
miR-505-3p and miR-19a-3p	Down-regulated in PCa cell lines down	inhibits invasion and migration	Deactivation of TGF-β signaling via targeting SMAD2, and SMAD4	[77,78]
miR-671-5p	Up-regulated in metastatic and localized PCa tissue (vs. control)	promotes proliferation, migration, and invasion in vitro and in vivo	Targeting NFIA/CRYAB axis	[79]
miR-802	Down-regulated in PCa tissue	suppresses EMT, migration and invasion (in vitro) and delays xenograft tumor formation in vivo	Flotillin-2	[80]
miR-940	Down-regulated in PCa tissue	suppresses migration and invasion	Migration and invasion enhancer 1 (MIEN1)	[81,82,83]
	Up-regulated in serum (PCa vs. control) [81]	promotes osteogenic differentiation of human mesenchymal stem cells		
miR-3622a	Down-regulated in PCa tissue	inhibits EMT, progression, and dissemination of tumor cells in vitro and in vivo	ZEB1 and SNAI2	[84]

PTEN: phosphatase and tensin homolog, RECK: reversion-inducing cysteine-rich protein with Kazal motifs; PDCD4: programmed cell death protein 4, KLF: Krüppel-like factor, TRAF: tumor necrosis factor receptor-associated factor, NF-κB: nuclear factor κB, TGF-β: transforming growth factor β; TGIF2: TGF-β-induced factor homeobox 2, NFIA: nuclear factor I A, CRYAB: Alpha-B crystallin; TNIP1: TNF-α induced protein 3 interacting protein 1, SOCS1: suppressor of cytokine signaling 1.

**Table 2:** Preclinical evaluations of therapeutic approaches based on miRNA in PCa.

TargetedmiRNA	Therapy Type	Delivery Technology/Vector	Cell Lines	In Vivo	Delivery Route In Vivo	Results	References
miR-16	Mimic	Atelocollagen	22Rv1 Du145 PPC-1 PC-3M-luc	PC-3M-luc	i.v	Local tumor growth inhibition in bone and downregulation of CDK1 and CDK2	[111]
miR-21	Anti-miR	Modified locked nucleic acid (LNA), poly nucleic acid (PNA)	DU145 PC3	DU145	i.v	Inhibition of PTEN and cell proliferation	[106]
						Reduced bone metastasis	
miR-34a	Mimic	Chitosan nanoparticles	PC3	PC3	i.v	Induction of autophagy and apoptosis by downregulation of MET and Axl and c-Myc	[112]
						Inhibition of tumor growth and metastasis preservation of bone integrity in vivo	
miR-124	Mimic	PEI functionalized polyhydroxybutyratenanoparticles (PHB-PEI)	PC3	-	-	Inhibition of proliferation, motility, and colony formation, through CPT1A modulation	[113]
miR-133a-3p	Mimic	Cationic-lipid transfection (Lipofectamine™ 3000)	VCaP C4-2B PC3	PC3	i.v	Activation of PI3K/AKT pathway and downregulation of EGFR, FGFR1, IGFR1	[114]
						Reduced tumor spread and bone metastasis in vivo	
miR-141 & miR-375	Mimic	cationic polymer polyethyleneimine (PEI) nanoparticles	LNCaP PC3 DU145	PC3	i.p	Reduced cell proliferation and increased apoptosis	[115]
						Tumor growth inhibition and up-regulation of SEC23A and PHLPP1 in vivo	
miR-143	Mimic	cationic polymer polyethyleneimine (PEI) nanoparticles	PC3 DU145	PC3	i.p	Tumor growth inhibition and decreased UPAR protein levels	[116]
miR-145	Mimic	PEI-modified magnetic nanoparticles with R11 peptide coating (R11-SSPEI)	PC3 LNCaP	PC3	i.p	Tumor growth inhibition and prolonged survival in vivo	[59]
miR-146a + cetuximab	Mimic	miR-146a and cetuximab-loaded nanocomplexes (PEI-PBA-miR-146a and PEI-DMA-cetuximab)	DU145	DU145		EGFR silencing and reduced cell growth, invasion, and migration in vitro	[117]
						Tumor growth inhibition by enhanced chemosensitivity in vivo	
miR-155-5p	Mimic	Cationic-lipid transfection (Lipofectamine™ 2000)	PC3	-	-	Decreased cell migration and invasion by up-regulation of vimentin, N-cadherin, E-cadherin, β-catenin, MMP3 and MMP9	[118]
						Inhibition of SPOCK1	
miR-205 + docetaxel	Mimic	Magnetic nanoparticle-based platform with iron oxide core (PEI-PEG)	C4-2 PC3	-	-	Enhanced chemosensitivity and apoptosis	[119]
miR-217 + miR-181b-5p + taxan	Mimic	Transfection (DharmaFECT-1)	PC3 DU145	-	-	Enhanced chemosensitivity and apoptosis	[120]
						Downregulation of more than a thousand different transcripts, which were enriched for genes with cell proliferation and focal adhesion functions	
